# A Subcutaneous Multiparameter Sensor With Integrated Interstitial Fluid Pressure Measurement for Remote Heart Failure Monitoring

**DOI:** 10.1016/j.jacbts.2023.03.004

**Published:** 2023-04-24

**Authors:** Alexander M.K. Rothman, Hamza Zafar, Rachel Sandy, Carl Wright, Sandip Mitra, Leonard Ebah, Duha Ilyas, Prasanna Hanumapura, Shereen Sebastien, Abubaker Khalifa, Joseph Passman, Robert S. Schwartz

Heart failure (HF) affects 6.9 million patients in the United States with 700,000 deaths per year. Following an index admission, hospitalization occurs in 24% of patients at 30 days. Despite improvements in medical therapy and the development of services specifically designed to reduce occurrence, the rate of rehospitalization remains high.

Volume overload, and the development of hemodynamic and clinical congestion drive symptoms and hospitalizations in patients with HF. Because of altered cardiac physiology and capillary Starling forces in patients with HF, the interstitial compartment and lymphatic system are disproportionately large reservoirs of fluid accumulation, increasing up to 3 to 4 times that of the intravascular compartment.^1^ The evaluation and management of fluid status is critical to management; however, the standard of care is based on the assessment of clinical signs and symptoms of congestion rather than evaluation of cardiac filling pressure or interstitial fluid volume. A transcatheter-implanted pulmonary artery pressure monitor may be used for outpatient hemodynamic monitoring of patients with HF and has been shown to reduce hospitalizations. Limitations to widespread adoption include the requirement for an invasive, cardiac catheterization–based implantation, limited reimbursement, and a device cost of approximately $20,000. In addition, the requirement for patient-activated measurements results in only 60% to 80% of patients providing weekly readings.^2^ Low measurement compliance impairs the collection of the longitudinal (trending) data, which, in turn, limits the availability of information on which to make therapeutic decisions. Alternatively, multiparameter measurements derived from implanted cardiac resynchronization therapy defibrillator devices provide a means to continuously monitor patients and identify those with an increased risk of HF hospitalization, without a requirement for patient-activated measurements.^3^ There is no device that combines absolute, hemodynamic data alongside multiparameter measurements.

Because of disproportionate reservoir capacity of the interstitial space, measurement of interstitial pressure is attractive for the evaluation of fluid status. In patients with HF, interstitial fluid pressure (IFP) correlates with indicators of impaired cardiac function, including reduced cardiac output and increased right atrial pressure, and the point of transition from sub-atmospheric to supra-atmospheric IFP is marked by rapid accumulation of interstitial fluid.^4^ As such, IFP is a quantitative, absolute, and physiologically distinct indicator of fluid status in a fluid compartment with disproportionate pathological importance. Because IFP is measured in subcutaneous tissue, this metric could be combined into the form factor of devices used for subcutaneous implantable cardiac monitors (ICMs), enabling the simultaneous measurement of multiparameter and hemodynamic data.

**Figure 1:**
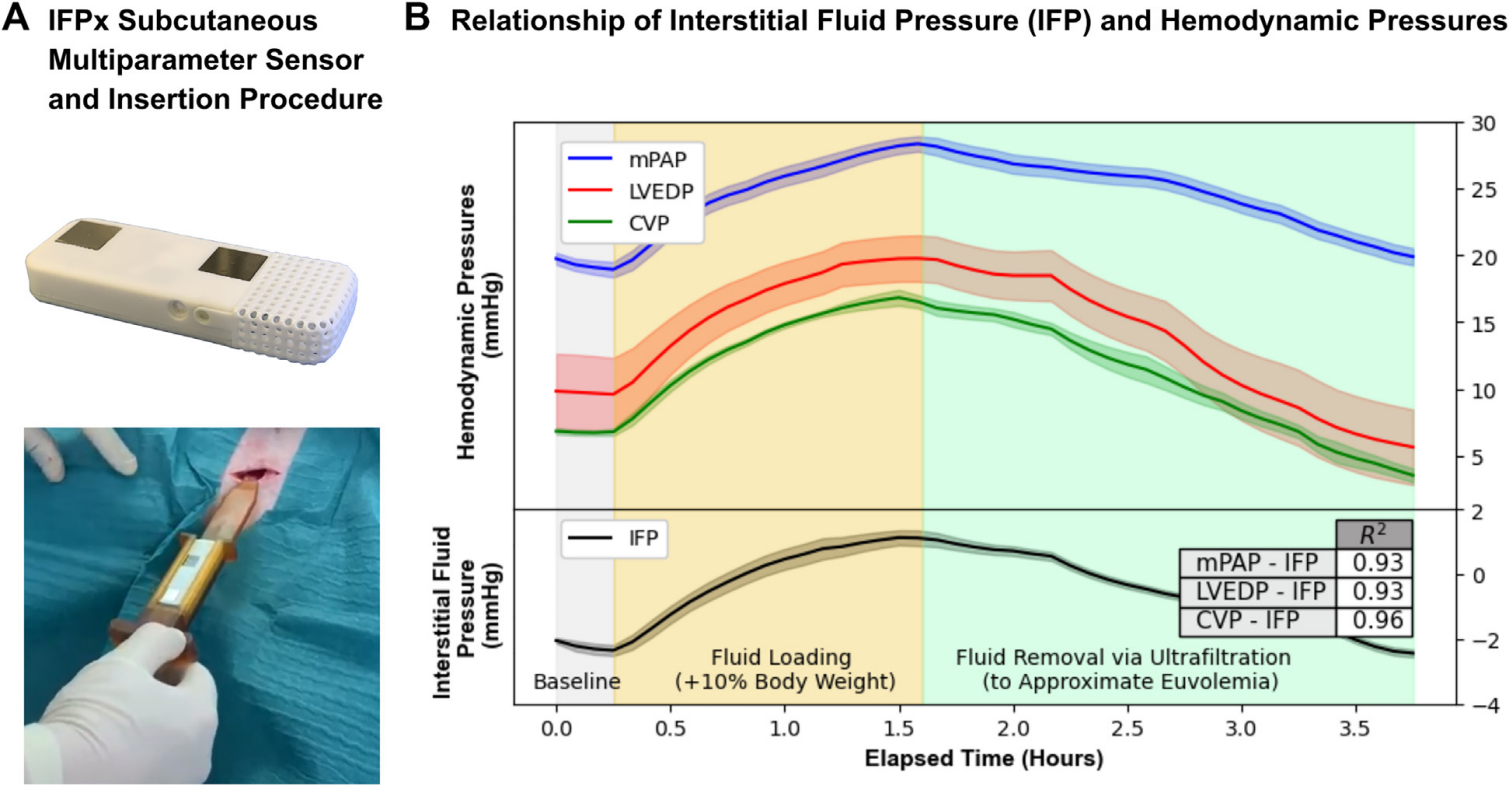
IFPx Sensor Demonstrates Relationship Between IFP and Hemodynamic Pressures in a Porcine Model of Acute Fluid-Overloaded Heart Failure **(A)** The IFPx sensor is inserted subcutaneously in a 5-minute procedure in an office-based setting under local anesthesia. The IFPx sensor measures interstitial fluid pressure (IFP), heart rate, activity, sleep incline, respiratory rate, and more. **(B)** Pilot data (5-minute rolling mean and SD, representative data, thoracic location) showing the relationship between IFP and hemodynamic pressures in a porcine model of acute fluid-overloaded heart failure. CVP = central venous pressure; LVEDP = left ventricular end diastolic pressure; mPAP = mean pulmonary artery pressure.

NXT Biomedical is developing IFPx, a minimally invasive remote monitor that adds IFP to the multiparameter data offered by ICMs (Figure 1A). This technology is based on the pioneering work of Arthur Guyton,^5^ who implanted a perforated capsule into the subcutaneous tissues of dogs to measure IFP. The original capsules were celluloid and methacrylate and contained approximately 100 holes of 1-millimeter diameter. The air inside the capsule was replaced with in-growing tissue and interstitial fluid 2 to 3 weeks after implantation. By inserting a needle into the capsule, Guyton^5^ was able to directly measure IFP and demonstrated negative interstitial pressures in certain tissues.

We have performed pilot studies with classic Guyton capsules that have shown a relationship between hemodynamic pressures and IFP in acute porcine models of fluid overload (Home Office License: PP1785781). Briefly, under general anesthetic, Yorkshire pigs were implanted with Guyton capsules and housed for 14 days. Under terminal anesthetic, animals were given 12 to 16 mg/kg per hour of a cardio-selective β_1_-blocker (esmolol, intravenously) and 0.9% NaCl (10% body weight/3 hours, intravenously) until a central venous pressure of >15 mm Hg was maintained. Fluid was then removed to euvolemia via ultrafiltration (3 hours). Concurrent measurement of IFP using the Guyton capsule and invasive hemodynamics (central venous pressure, pulmonary artery pressure, and left ventricular end diastolic pressure) was made using standard interventional techniques. IFP, measured in the thoracic location, increased, and decreased with invasive hemodynamic measures during fluid loading and ultrafiltration, respectively (Figure 1B). As such, IFP is related to hemodynamic parameters and capillary filtrate formation.

The IFPx sensor is a is a minimally invasive, subcutaneously implantable device that has been developed to address deficiencies in current remote HF monitoring technology and provide quantitative, absolute fluid status assessment alongside the multiparameter data offered by insertable cardiac monitors. The IFPx device connects a chronically stabilized Guyton capsule to a commercial, digital, temperature-compensated pressure transducer and is inserted via a small incision in the subcutaneous tissue with an insertion tool, in a 5-minute procedure identical to that of commercial ICMs. The multiparameter dataset includes IFP, heart rate, physical activity, and sleep incline and is relayed to a cloud-based digital health system without patient interaction. Furthermore, the IFPx device is removable in the same fashion as ICMs. The IFPx device will facilitate the provision of proactive, individualized treatments for patients with conditions of significant fluid overload, including HF.
